# Psychopathology in female offenders of terrorism and violent extremism: a systematic review

**DOI:** 10.3389/fpsyt.2023.1123243

**Published:** 2023-06-27

**Authors:** Sadaf Rakhshandehroo, Nils Duits, Dick Bergman, Robbert-Jan Verkes, Maaike Kempes

**Affiliations:** ^1^Department of Science and Education, Netherlands Institute of Forensic Psychiatry and Psychology, Utrecht, Netherlands; ^2^Donders Institute for Brain, Cognition and Behaviour, Radboud University, Nijmegen, Netherlands; ^3^Department of Criminal Law and Criminology, Radboud University, Nijmegen, Netherlands; ^4^Faculty of Social and Behavioural Sciences, Leiden University, Leiden, Netherlands

**Keywords:** psychopathology, mental disorder, terrorism, violent extremism, systematic review

## Abstract

**Introduction:**

Terrorism and violent extremism are major social threats worldwide and are committed not only by men but also by women. Previous research has shown indications of psychopathology, among other personal and contextual factors, as a potential risk factor for perpetrating terrorist and violent extremist crimes. Despite the fact that women have engaged in acts of terrorism and violent extremism throughout history, the vast majority of literature on psychopathology so far has been mainly focused on men with terrorist and violent extremist behavior. As women's engagement in terrorism and violent extremism is increasing, and gender differences in psychopathology in offenders of terrorism or violent extremism may exist based on empirical evidence for such differences in offenders of violence, gender-informed research into psychopathology as a potential risk factor for offending is of pivotal importance for improving the effectiveness of counter-terrorism interventions. The present systematic review was designed to examine what empirical knowledge exists on the presence and potential contributing role of psychopathology in female perpetrators of terrorism or violent extremism.

**Methods:**

A literature search was conducted to identify primary source studies in PsycINFO, PubMed, Embase, Web of Science, and Sociological Abstracts. ASReview as an artificial intelligence software was used to screen references.

**Results:**

In total, eight studies were included, of which only two studies distinguished prevalence rates and types of psychopathology separately for women, indicating personality disorder as most common. All four out of the eight studies that reported on the relationship between psychopathology and terrorism and violent extremism assumed psychopathology to be a contributing factor in engaging in terrorist or violent extremist acts. However, none of these four studies reported on potentially present female-specific mechanisms of the role of psychopathology in offenses.

**Discussion:**

The present systematic review draws the striking conclusion that there is a lack of clearly described empirical studies on psychopathology in female perpetrators of terrorism and violent extremism and emphasizes the importance of more future empirically based inquiries on this topic by the forensic psychiatric field.

**Systematic review registration:**

https://www.crd.york.ac.uk/prospero/display_record.php?RecordID=275354, identifier: CRD42021275354.

## 1. Introduction

Terrorism and violent extremism are major social threats worldwide. In 2021, the number of attacks globally rose by 17% since 2015 and led to more than 7,000 deaths (1). As terrorism and violent extremism pose an enormous threat to citizens and societies, countering terrorism and violent extremism remains an ongoing global security challenge (2). To counter terrorism and violent extremism more effectively, more evidence-based knowledge about personal and contextual risk factors contributing to terrorism and violent extremism engagement is needed (3–6). Psychopathology, in particular, is one of the potential risk factors associated with “grievance-fueled targeted violence” and more research into its relationship with acts of terrorism and violent extremism is required (7, p.459, 8).

For readability purposes, we clarify here what is meant by terrorism, violent extremism, and psychopathology in the present paper. In the literature, many definitions and types of terrorism exist, with a tendency to equate radicalism with terrorism and extremism (9–11). Although some radicalized persons engage in terrorist crimes, not every radicalized individual commits a terrorist act (9). In this article, we have used the following definition for terrorist act: “The threat, preparation or perpetration of serious violence based on ideological motives against people, or deeds aimed at causing socially disruptive material damage, with the goal to cause social change, to instill fear among the population or to influence political decision-making” (12). Regarding violent extremism, we used the following definition: “The unlawful use of force and violence against persons or property to intimidate or coerce a government, the civilian population or any segment thereof, in furtherance of political or social objectives” (13). Concerning psychopathology, we defined this in our study as mental disorders, pathological personality traits, symptoms or psychological problems. For mental disorders, we used the following definition as stated in the Diagnostic and Statistical Manual of Mental Disorders, Fifth Edition, Text Revision (DSM-5-TR): “A syndrome characterized by clinically significant disturbance in an individual's cognition, emotion regulation or behavior that reflects a dysfunction in the psychological, biological or developmental processes underlying mental functioning” (14). Although psychopathology in populations with terrorist and violent extremist behaviors has been the subject of scientific research since the 1970s (15), empirical evidence for the common assumption that offenders of terrorism and violent extremism must be insane or psychopathic did not emerge until the early 2000 (15, 16). Since 2013, however, the number of studies based on empirical data on this matter has increased (17). This has resulted in a shift away from the binary thinking of as either mentally ill or not mentally ill, and toward an embracement of complex pathways consisting of an interplay of multiple factors such as criminal history, personal experiences, beliefs, and mental health, resulting in terrorist and violent extremist behavior (7, 18).

Regarding mental (dis)functioning, three systematic reviews on the prevalence of psychopathology in populations with terrorist or violent extremist behaviors have been published. In a systematic review by Gill and colleagues (19), it was concluded that prevalence rates of mental health problems in various groups of radicalized individuals and terrorist populations ranged from 0% to 57%. In another systematic review, Trimbur and colleagues (20) found prevalence rates of psychiatric disorders between 3.4% and 48.5% among different groups of terrorist populations. A recent Campbell systematic review (21) performed a meta-analysis of studies reporting psychological problems, disorders, and suspected disorders in samples with terrorist behaviors and measured a pooled prevalence rate of 25.5%.

As can be seen from the collected studies in the abovementioned systematic reviews, it is striking to note that most studies on psychopathology in terrorist and violent extremist populations seem to have been mainly focused on male perpetrators despite the fact that women have engaged in terrorist and violent extremist acts throughout history (22, 23). The role of female perpetrators of terrorist and violent extremist acts has largely been viewed as novel or less relevant by scholars (24) maybe because the number of women is relatively small compared to men in terrorist and violent extremist populations (25).

Particularly since the rise of the so-called Islamic State in 2014 and its active inclusion of women as perpetrators of terrorist acts, more women have engaged in terrorism (26, 27). According to a report by the International Center for Counter-Terrorism (28), women represented up to 16 percent of foreign nationals who traveled to regions of the Islamic State in Iraq and Syria. The return of these women to their countries of residence has concerned policymakers of these countries considering the potential threat that may emanate from these women (29, 30). Because psychopathology, among other personal and contextual factors, is considered a potential risk factor for perpetrating terrorist and violent extremist acts (8, 31), gaining insight into the psychic (dis)functioning of female perpetrators of terrorism and violent extremism is of pivotal importance.

In contrast to the apparent lack of studies on psychopathology in female populations with terrorist and violent extremist behaviors, this topic has been better studied in female non-terrorist offenders of violence, and differences in types of psychopathology between female and male offenders of violence have been found. More specifically, De Vogel and colleagues (32) found that women with violent offenses were more often diagnosed with depression, posttraumatic stress disorder, and borderline personality disorder whereas men were more likely to be diagnosed with addiction, sexual disorder, and narcissistic and antisocial personality disorders. Another study (33) showed that female offenders of violence were more often diagnosed with depression and traits of borderline personality disorder, were more likely to have been sexually and physically abused and emotionally neglected, showed more self-harming behaviors, and had lower self-esteem. Gender specific differences in psychopathy were found in a study by Klein Haneveld and colleagues (34). Within this study, thresholds in specific items of the Psychopathy Checklist Revised (PCL-R), i.e., grandiose sense of self-worth, poor behavioral controls, promiscuous sexual behaviors, and many short-term marital relationships were found to be gender variant.

Besides existing knowledge on prevalence rates and types of psychopathology in women with violent behaviors, studies also exist on the relevance of psychological problems for such behaviors. For example, a study by Warren and colleagues (35) showed that cluster B personality disorders, e.g., antisocial and narcissistic personality disorder play a role in violent acts by women. Other studies showed that depression and suicidality play a part in filicide by women (36, 37). On the potential role of mental health problems in women with terrorist or extremist behaviors some indications have been found by Jacques and Taylor (38) who, based on open source data, investigated female suicide bombers showing that they were seemingly more motivated by feelings of unhappiness or suicidal thoughts. Interestingly, statistically significant differences were found with male suicide bombers who had apparently been more driven by extreme religious or nationalistic ideas.

Given the occurrence of psychopathology in men with terrorist and violent extremist behaviors, the potential gender differences herein, and the fact that the results of the existing systematic reviews on this topic are not gender-specified, the present systematic review was designed to fill this void by examining what empirical knowledge exists on presence and relevance of psychopathology in female perpetrators' acts of terrorism or violent extremism. While conducting this systematic review, we also attempted to address the following limitations generally seen in research on psychopathology in terrorist and violent extremist populations.

First, as a considerable amount of the available studies on psychopathology in terrorist or violent extremist populations lacks empirical evidence and is based on secondary source data (16, 39), e.g., media, instead of primary sources, i.e., psychological or psychiatric examinations, we included only studies based on primary source data. Furthermore, by taking into consideration mental disorders in addition to pathological personality traits, symptoms, and psychological problems, we aimed to avoid the previously established dichotomous approach of individuals with terrorist or violent extremist behaviors as either mentally ill or not mentally ill (7, 40, 41). In addition, we included only studies of offenders of terrorism and violent extremism, and thus excluded studies of non-offending radicalized persons due to the fact that not every radicalized individual engages in an act of terrorism or violent extremism (9). Finally, because of the importance of the potential contributing role of psychopathology in terrorist and violent extremist offenses (42), in addition to prevalence studies we also included studies addressing psychopathology as a contributing factor in acts of terrorism or violent extremism.

The present systematic review had two objectives, i.e., searching for existing empirical evidence for (a) psychopathology in female perpetrators of terrorism and violent extremism; and (b) its potential role in their crimes. In accordance with these objectives, the two following research questions were formulated: What is the empirical knowledge available about (1) the presence, prevalence rates, and types of psychopathology in female offenders of terrorism and violent extremism; and (2) the role of psychopathology as a potential contributing factor to female involvement terrorism or violent extremism?

## 2. Methods

### 2.1. Protocol and registration

For a consistent and transparent report of the results, this systematic review was conducted in accordance with the established systematic review guidance PRISMA (Preferred Reporting Items for Systematic Reviews and Meta-Analyses) (43, 44). An a priori study protocol was prepared and registered in the international prospective register of systematic reviews PROSPERO (ID CRD42021275354).

### 2.2. Inclusion and exclusion criteria

The inclusion criteria were (1) original studies comprising case reports or case series investigating the prevalence or relevance of psychopathology in adult female perpetrators of terrorism or violent extremism, (2) in which the study population was directly examined, i.e., through psychological or psychiatric examination, questionnaires or interviews, or indirectly examined based on data from primary sources, i.e., data from direct examination by health professionals or trained staff, and (3) written in English, Dutch, French, or German. Papers in other languages could not be incorporated due to language restrictions in our review team.

The exclusion criteria were (1) non-original publications, e.g., reviews, commentaries, viewpoints, editorials, theoretical papers with no data from psychiatric assessment of subjects, and (2) studies addressing the psychopathology of radicalized individuals who had not committed acts of terrorism or violent extremist.

### 2.3. Outcome measures

In this mixed quantitative and qualitative systematic review, three outcome measures were formulated. Quantitative outcomes included prevalence rates and types of psychopathology. The qualitative outcome was a description of the relevance of psychopathology, i.e., mechanisms of its influence or role in terrorist or violent extremist acts.

### 2.4. Search strategy

A literature search was conducted on September 28, 2021 for studies up to that date, in the electronic bibliographic databases PsycINFO (Ebsco), PubMed, Embase, Web of Science, and Sociological Abstracts. On May 30, 2022, the literature search was performed again in order to look for studies published in the period between the initial search and that date. In the online Supplementary material, the search terms for our literature search are included. The entire search strategy and the references per database can be requested from the corresponding author.

To identify published studies that we did not come across via the search string, citation tracking was used. Furthermore, gray literature, i.e., information produced by government agencies, academic institutions or other sectors that is not typically made available by commercial or academic publishers, was included in the search.

### 2.5. Screening strategy for identifying relevant studies

For the screening phase, an open source machine learning aided software with state-of-the-art active learning techniques for systematic reviews called ASReview (Active learning for Systematic Reviews) was used (45). Versions 0.18 and 0.19 of ASReview were used for the initial and second search, respectively. The decision to use artificial intelligence rather than the usual manual method used for systematic reviews is described in detail in the online Supplementary material.

In order to train ASReview on which studies were of interest and which were not, examples of relevant and irrelevant publications known to the authors prior to conducting the current review were first entered into this software program. Based on this information, ASReview ranked all references from the most to the least relevant and put the most relevant reference at the top of the list to be screened first by the two reviewers responsible, i.e., the first and third author. The reviewers screened the references independently from one another, based on title, abstract, and full text. The reason why full texts were already read at this stage of screening was to avoid publications being incorrectly scored as relevant or irrelevant, which would have misled the learning models. Only in case a publication could be identified with certainty as irrelevant on the basis of title and abstract, was the full text not read.

To ensure that the relevant references were found, three rounds per reviewer were conducted with each round having its own active learning model. There was no difference in the active learning models between the two reviewers. As a stopping rule within each round, i.e., to know when to stop screening, it was decided not to screen further after consecutively scoring one hundred references as “irrelevant”. In case only one “relevant” reference was found in the last 25% of the one hundred irrelevant references, screening could also be stopped. After each round, both reviewers let each other know whether, and if so, which relevant article(s) they had found. Any discrepancies in the relevance of a reference between the reviewers would first be resolved through a discussion between them. In case no consensus could be reached, a third researcher, i.e., the second author, could then be consulted whose judgment would be decisive.

More detailed descriptions of the rationale behind the use of an artificial intelligent software, the specifically chosen screening strategy, and the accessed learning models for this systematic review are included in the online Supplementary material.

### 2.6. Data extraction

After identifying the relevant studies, data extraction was performed by the two reviewers, i.e., the first and third author, independently of each other. Primarily, the choice was made to extract female-specific results followed by a comparison with results concerning men. In case a study did not report gender-specific data on psychopathology, the corresponding author was to be contacted via e-mail in order to see whether data on women could be provided. In case the majority of the included studies still lacked female-specific data, the “mixed” results, i.e., those reported for female and male subjects taken as one group, were to be extracted. If studies under review included a relatively low number of subjects, prevalence rates would primarily be presented in terms of the number of subjects followed by percentages throughout the review to provide a more nuanced representation of the prevalence rates.

### 2.7. Assessment of the risk of bias in the included studies

The methodological quality of the included studies was measured to better interpret the validity of their reported results. For this purpose, the Scottish Intercollegiate Guidelines Network (SIGN) framework was used (46). The levels of evidence according to SIGN are included in the online Supplementary material.

In addition to using the SIGN framework, the quality of each study under review was also evaluated by us on the basis of its selection of assessment tool(s), and the differentiation of results between female and male participants. These aspects were specifically chosen since use of primary sources and data specifically about women were a particular focus of our review.

## 3. Results

### 3.1. Literature search results

**Figure 1:**
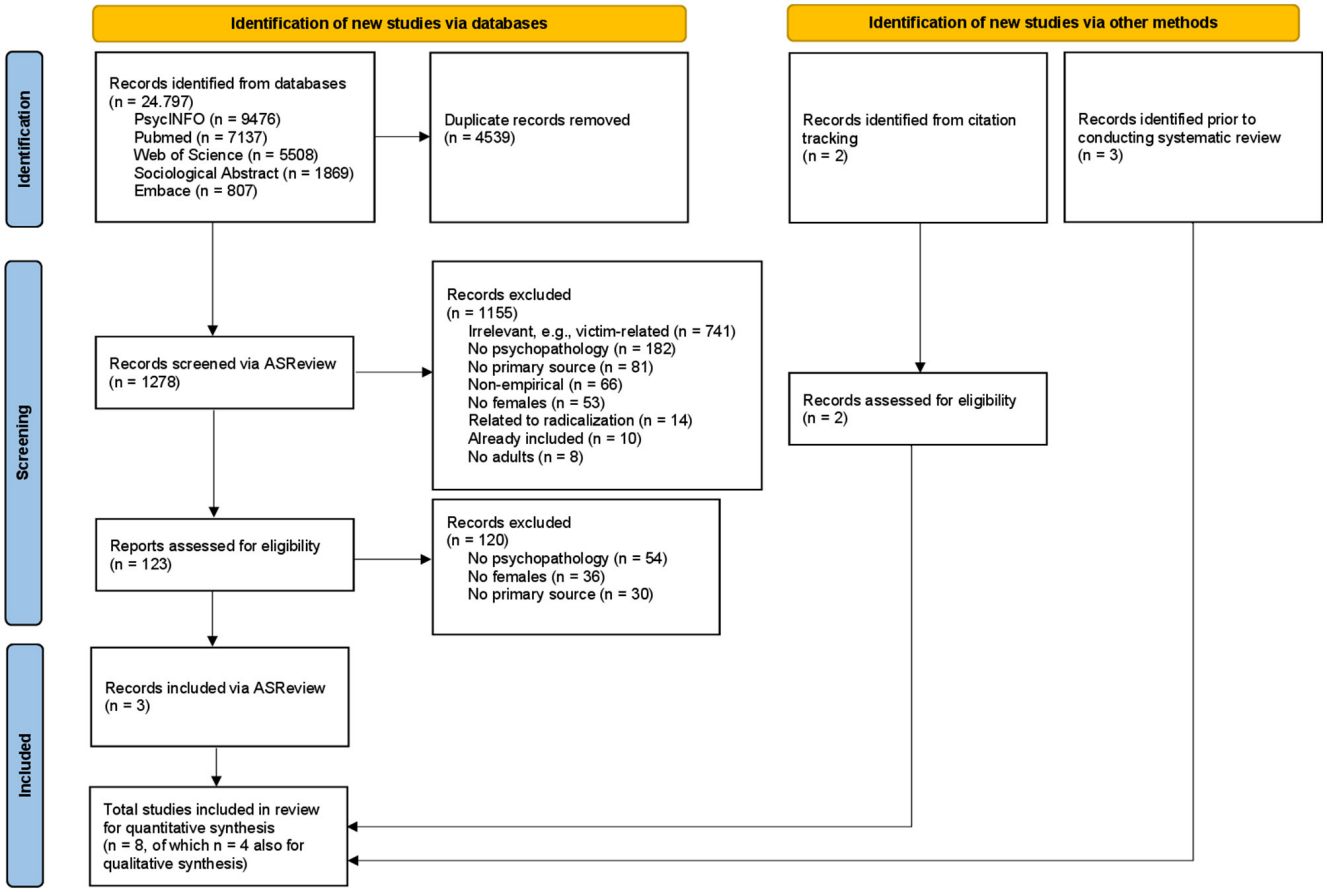
PRISMA flow diagram of study selection process for systematic review.

As shown in the PRISMA flow diagram (Figure 1), the initial and second literature search resulted in 20,258 records after removing the duplicate records. Using ASReview, 1,278 references were screened. In total, eight studies met our inclusion criteria and were included in this systematic review. Three of the eight studies were found via ASReview after both searches (8, 47, 48) whilst two other relevant studies were found via citation tracking after the initial search (49, 50). Furthermore, two relevant studies were known to the authors of the review prior to conducting the initial search (51, 52), and one study (53) was published after the initial search and known to the authors of the review prior to the second search. Consensus on all inclusions was reached between the two reviewers without having to consult the third researcher.

### 3.2. Characteristics of included studies

The characteristics of the included studies are shown in Table 1. The total number and proportion of women in the included studies compared to men could not be measured because in one study (50) on non-ideologically inspired murderers, no distinction was made in the numbers of female (or male) subjects between the study group and the control group. All eight studies were cross-sectional and eligible for quantitative analysis regarding prevalence rates and types of psychopathology. For the qualitative synthesis concerning the role of psychopathology in acts of terrorism or violent extremism four of the eight studies were eligible (8, 47, 49, 50).

**Table 1:** Description of review studies and prevalence rates of psychopathology.

**References**	**Country**	**Study group's characteristics**	**Control group's characteristics**	**Terrorist or violent extremist acts**	**Assessment tool(s)**	**Female-specific prevalence rates [male-specific prevalence rates]**	**Mixed prevalence rates for females and males [in control group]**	**Level of evidence based on SIGN**
Bubolz and Simi (49)	United States	Former members of violent white supremacist groups. *N =* 44 Female: 6 (14%); 19–61 years old Male: 38 (86%); 19–61 years old	None	Membership in violent white supremacist groups	Life-history interviews with structured and unstructured components	None	Mental health problems preceding or during extremist involvement: *N =* 25 (57%)	3
Duits et al. (8)	The Netherlands	Jihadist terrorism offenders. *N =* 118 Female: 5 (4%); ≥ 22 years old Male: 113 (94%); ≥ 22 years old	None	Threat, preparation or perpetration of violence based on ideological motives against people, or deeds aimed at causing socially disruptive material damage to cause social change, instill fear or influence political decision-making	VERA-2R	None	Mental disorder(s): *N =* 33/73^a^ (45%)	2^−^
Gill et al. (51)	United kingdom	Lone-actor Jihadists and right-wing inspired terrorists. *N =* 49 Female: 6 (12%); Age not mentioned Male: 43 (88%); Age not mentioned	None	Engaged in or planned to engage in lone-actor terrorism. Females all Jihadists	Psychological assessments	None	Mental illness: *N =* 16 (33%)	3
Lyons and Harbinson (50)	Northern Ireland	Politically motivated murderers in jail. *N =* 47 Female^b^: 8 (7%); 32 years old Male^b^: 98 (93%); 32 years old	Non-ideologically inspired murderers in jail. *N =* 59 Female^b^: 8 (7%); 32 years old Male^b^: 98 (93%); 32 years old	Directly involved in a violent act (e.g., pulling the trigger or planting bombs)	Interviews, case notes, and hospital records	None	Mental illness: 16%^c^ [58%^c^]	3
Merari and Ganor (47)	Israel	Palestinian lone-actors. *N =* 27^d^ Female: 5 (19%); 18–39 years old Male: 22 (81%); 18–38 years old	None	Religiously-inspired lone-actor terrorists who conducted independent attacks (i.e., attacks which were performed with no organizational involvement in their conception, planning and execution)	Semi-structured clinical psychological interviews, Rorschach test, MMPI-2, SCID 5 PD, SCID 5 SPQ, TAT	Psychological problem(s) or mental disorder(s): *N =* 4 (80%) [*N =* 13 (59%)]	Psychological problem(s) or mental disorder(s): *N =* 17 (63%)	3
Morris and Meloy (48)	Scotland UK	Subjects posing a national security risk. *N =* 23 Female: 1 (4%); 23–72 years old Male: 22 (96%); 23–72 years old	None	Criminal acts and expressions of concern about potential vulnerability to involvement in terrorist activities expressed by friends, family or professionals	HCR-20, Structural Professional Judgement tool	Abnormal personality structure: *N =* 1 (100%) [Pathological personality traits: *N =* 7 (30%)]	History of psychiatric disorder, leading to contact with mental health services: *N =* 9 (39%)	3
Thijssen et al. (53)	The Netherlands	Convicts of terrorism in detention. *N =* 82 Female: 7 (9%); 20–34 years old Male: 75 (91%); 19–50 years old	None	Related to jihadist (majority) or right-wing extremist groups	Psychological evaluations by psychologists, medical doctors, nurses, and, if applicable, a psychiatrist	None	Psychiatric disorder(s): *N =* 26 (32%)	3
Van Leyenhorst and Andreas (52)	The Netherlands	Convicts of terrorism. *N =* 26 Female: 4 (15%); Mean age: 21 years Male: 22 (85%); Mean age: 21 years	None	Convicted of terrorism-related offenses connected to Salafi-Jihadi terrorism (e.g., foreign fighter, intent to join, recruit support or logistic support for terrorism related activities)	Forensic pre-trial diagnostic reports	None	Mental disorder(s): *N =* 4 (15%)	3

HCR-20, Historical Clinical Risk Management−20; MMPI-2, Minnesota Multiphasic Personality Inventory 2; SCID 5 PD, Structured Clinical Interview for DSM 5 Personality Disorders; SCID 5 SPQ, Structured Clinical Interview for DSM 5 Screening Personality Questionnaire; SIGN, Scottish Intercollegiate Guidelines Network; TAT, Thematic Apperception Test; VERA-2R, Violent Extremism Risk Assessment 2 Revised.

^a^Of whom with available data on psychopathology; ^b^Study and control group taken together; ^c^N not mentioned by authors of the current review due to illogical calculation in this specific study; see this study for more information; ^d^N of adult individuals within this study.

Two studies (8, 47) included female samples consisting of a mix of adult and juvenile participants. In the study by Duits and colleagues (8), “adult” women were defined as those aged between 22 and 60 years, which we included in our review in accordance with our inclusion criterion, i.e., adults. In the study by Merari and Ganor (47), women aged 18 years or older were included in our review.

The most recent of the studies under review was by Duits and colleagues and was published in 2022 whilst the oldest was by Lyons and Harbinson, published in 1986. The countries of origin were Northern Ireland, the Netherlands, Scotland, the United Kingdom, and the United States. One study included a control group (50). In two studies, the description of the participants met our definition of violent extremists (49, 50) whereas in the remaining six studies, the description met our definition of terrorists. The answers to the two research questions follow below, under Sections 3.3 (first research question) and 3.4 (second research question).

### 3.3. Prevalence rates and types of psychopathology in offenders of terrorism and violent extremism

Regarding the first research question, i.e., what empirical knowledge is available on presence, prevalence rates, and types of psychopathology in female perpetrators of terrorism and violent extremism with the exception of two studies (47, 48), the remaining six studies reported “mixed” prevalence rates and types of psychopathology, i.e., for women and men considered as one group. We first summarize the “mixed” findings given in the studies under review (Section 3.3.1) followed by the findings of the two studies (47, 48) that distinguished female-specific prevalence rates and types of psychopathology (Section 3.3.2).

#### 3.3.1. “Mixed” prevalence rates and types of psychopathology in offenders of terrorism and violent extremism

First, the “mixed” prevalence rates of non-specific mental disorders, pathological personality traits, symptoms and psychological problems are presented (Section 3.3.1.1). Next, the “mixed” prevalence rates of specific mental disorders (Section 3.3.1.2) and specific pathological personality traits, symptoms, and psychological problems follow (Section 3.3.1.3). For clarity, these prevalence rates and types of psychopathology refer to both female and male participants, and women-specific data remain unclear as these six studies do not report on how many women were in each subgroup with a particular psychopathology.

##### 3.3.1.1. Mental disorders, pathological personality traits, symptoms or psychological problems in general

The prevalence of mental disorders, pathological personality traits, symptoms or psychological problems in general varied between 15% and 67% (see Table 1). The studies have used different terminology for describing psychopathology, e.g., “mental disorder” (8, p. 8), “history of psychiatric disorders” (48, p. 1641) or “psychopathological problems” (47, p. 7). One study (50) compared politically and non-politically motivated murderers, and concluded that the former were less likely to have a mental illness than the latter (see Table 1). This conclusion, however, needs to be considered with caution due to a discrepancy as the authors report there to be 60 non-politically motivated murderers (resulting in a prevalence rate of mental illness of 59%) whilst there were only 59 offenders in this comparison group. Contacting the authors of this study (from 1986) to gain more information on this discrepancy was not possible because no contact information could be found.

##### 3.3.1.2. Specific mental disorders

Seven of the eight studies under review measured the prevalence rates of specific mental disorders (8, 47, 48, 50–53). The different types of mental disorders and their prevalence rates are presented in Table 2. Personality disorders were reported in all seven studies with varying prevalence rates between 2% and 44%. Cluster B personality disorders were the most common, however, cluster A and C personality disorders also occurred (8, 48, 53).

**Table 2:** Prevalence rates of specific mental disorders in review studies.

**References**	**Intellectual disability**	**ADHD**	**ASD**	**PTSD**	**Anxiety disorder**	**Psychotic disorder**	**Mood disorder**	**Substance use disorder**	**Personality disorder**	**Neurocognitive disorder**	**Gambling disorder**
**N (%)**											
Duits et al. (8)	9 (12%)	1 (1%)	1 (1%)	2 (3%)	1 (1%)	7 (9%)	4^a^ (5%)	12 (6%)	8 (11%)	2 (3%)	-
Gill et al. (51)	2 (4%)	-	-	-	-	5^b^ (10%)	6 (12%)	-	1 (2%)	-	-
Van Leyenhorst and Andreas (52)	-	4^c^ (15%)	4^c, d^ (15%)	4^c^ (15%)	-	4^c^ (15%)	-	-	4^c, e^ (15%)	-	-
Lyons and Harbinson (50)	-	-	-	-	-	-	-	-	(36%)^f^	-	-
Merari and Ganor (47)	-	-	-	-	-	-	-	-	8 (30%)	-	-
Morris and Meloy (48)	-	-	-	-	2 (22%)	2 (22%)	3^1^ (33%)	6 (67%)	4 (44%)	-	-
Thijssen et al. (53)	7 (9%)	1 (1%)	5 (6%)	2 (2%)	-	6^g^ (7%)	2^h^ (2%)	10^i^ (12%)	5 (6%)	-	1 (1%)

ADHD, Attention Deficit and Hyperactivity Disorder; ASD, Autism Spectrum Disorder; PTSD, Posttraumatic Stress Disorde.

^a^Depressive disorder; ^b^Schizophrenia; ^c^Prevalence of ADHD, psychotic disorder, borderline personality disorder, PTSD, and ASD all taken together; ^d^Pervasive Developmental Disorder, Not Otherwise Specified (PDD NOS); ^e^Borderline personality disorder; ^f^N not mentioned by authors of the current review due to illogical calculation in this specific study; see this study for more information; ^g^Of whom one diagnosed with schizophrenia; ^h^Of whom one diagnosed with bipolar I disorder and one with major depressive disorder; ^i^Of whom two diagnosed with alcohol use disorder and eight with cannabis use disorder.

##### 3.3.1.3. Specific pathological personality traits, symptoms or psychological problems

Some studies included in this systematic review addressed specific pathological personality traits, symptoms, or psychological problems among subjects.

Suicidal thoughts or attempts were reported in two studies (47, 49), i.e., in 27 (62%) of the former members of violent white supremacist groups (49), and in 11 (41%) of the Palestinian lone-actors (47).

Symptoms of problematic substance use were reported in five of the eight studies (8, 47, 49–51). In former members of violent white supremacist groups, 32 (73%) subjects self-reported problems with alcohol or drugs (49). A history of substance abuse was found to be present in 11 (22%) of lone-actor Jihadists and right-wing inspired terrorists (51). Alcohol related problems and alcohol dependence were measured in 49% of a sample consisting of politically and non-politically motivated murderers (50). Within this study, the lower prevalence of committing murder under the influence of alcohol among politically motivated murderers (10%) vs. non-politically motivated murderers (57%) needs to be considered with caution due to the earlier mentioned discrepancy in the total number of the non-politically motivated murderers. In Palestinian lone-actors, two (8%) subjects had a record of drug or alcohol abuse (47). Symptoms of substance use were measured in 20 (27%) Dutch terrorist offenders (8). Within this population, other specific pathological personality traits, symptoms or psychological problems were also present including (with a prevalence rate of 20% or higher) problems with relationships (*N* = 24, 32%), poor regulation of aggression (*N* = 15, 20%), and distrust or paranoid feelings (*N* = 15, 20%).

Other specific psychological problems mentioned in the studies were deviant sexual behavior or interest in 14 (29%) lone-actor Jihadi or right-wing inspired terrorists (51) and psychotic background in 5 (23%) Palestinian lone-actors (47).

#### 3.3.2. Female-specific prevalence rates, types and role of psychopathology in female perpetrators of terrorism and violent extremism

Two of the eight studies under review reported female-specific data on the prevalence of psychopathology (47, 48). The authors of both studies were successfully contacted via e-mail in order to obtain more detailed information on the female-specific data within their studies. Of the remaining six studies, the mailing address of the corresponding author of five studies [except for Lyons and Harbinson, (50)] was available so that the author could be contacted via e-mail and asked for women-specific data. A response was received from the corresponding author of four studies (8, 51–53) on the basis of which we came to know that women-specific prevalence rates had not been determined separately due to the low number of female subjects.

In the study by Merari and Ganor (47), suicidality was present in three (60%) women whilst psychotic background or substance abuse were not present.

The only female participant in the study by Morris and Meloy (48) had refused psychiatric examination. Nevertheless, based on the available information, i.e., police reports and social work reports, she was suspected to have a problematic “personality structure as opposed to an affective or psychotic disorder” (48, p. 1642).

### 3.4. Psychopathology as a contributing factor to participation in acts of terrorism or violent extremism

To answer the second research question, i.e., what empirical knowledge is available on psychopathology as a potential contributing factor to female involvement in terrorism or violent extremism, the results of the four out of eight studies that reported on this topic are described here (8, 47, 49, 50). None of these four studies made a gender distinction in the role of psychopathology in acts of terrorism or violent extremism.

Based on the study of Dutch offenders of terrorism (8), cluster B personality traits, i.e., narcissistic and antisocial personality traits, were associated with perceived injustice contributing to making individuals more prone to terrorism. In the study of Palestinian lone-actor terrorists who were religiously inspired (47), mental health problems were found to be causally involved in the decision to carry out an attack. According to this study, the purpose of the attack was generally “to die or to be arrested, in order to escape from an intolerable life” (47, p. 19). Another study that used life-history interviews with former members of violent white supremacist groups found that “individuals with a history of mental health problems may have been disproportionately rejected by conventional social networks and, thus, drawn toward marginalized social groups such as an extremist organization” (49, p. 8). Lastly, the study on the role of problematic substance use within a group of politically and non-politically motivated murderers concluded that the relationship between problematic substance use and aggression was “a complex one where pharmacological, developmental, personality, and situational considerations all play a part” (50, p.196).

Regarding the role of psychopathology in terrorist and violent extremist acts, female-specific results were not given in any of the four studies that covered this topic (8, 47, 49, 50).

### 3.5. Quality of included studies

The quality assessment of the studies under review as measured by SIGN showed an overall low to moderate quality of evidence (see Table 1). The additional quality assessment by reviewers based on each study's selection of assessment tool(s) and distinction in results between female and male participants is as follows.

Regarding the assessment tools, the majority of the studies used high quality data sources, e.g., psychological or psychiatric tests, examinations, reports or structural professional judgment (8, 47, 48, 50–53). In one study, participants self-reported mental health problems (49). With respect to a distinction in results between female and male participants, it was remarkable to note that only two of the eight studies made a distinction in gender in prevalence rates of psychopathology (47, 48). Furthermore, none of the studies reported on the gender-specific role of psychopathology in acts of terrorism or violent extremism. The non-reporting of female-specific results on presence and relevance of psychopathology is considered as a major weakness of most studies under review.

## 4. Discussion

With the present systematic review, we aimed to shed light on psychopathology in offenders of terrorism and violent extremism. To achieve this goal, we searched for empirical, primary source literature on the presence and contributing role of psychopathology in crimes within this population. This systematic review revealed that only a few studies have investigated psychopathology in female perpetrators of terrorism and violent extremism, and that an even smaller number of these studies differentiate results between women and men. Nonetheless, the results show that psychopathology does exist in this population, and might play a contributing role in terrorist or violent extremist behavior.

Our first research question was to examine the existing empirical knowledge on presence, prevalence rates, and types of psychopathology in female perpetrators of terrorism and violent extremism. Of the eight included studies on this topic, two studies reported female-specific data (47, 48), and showed that pathological personality traits, personality disorders, and suicidality were prevalent. Due to this limited data, however, no conclusions can be drawn about prevalence rates and types of psychopathology in women. The ‘mixed' prevalence rates, i.e., measured within groups consisting of both women and men, vary in a wide range and are roughly consistent with the findings of the existing systematic reviews on this topic (19–21). An explanation for the variations in the prevalence rates of psychopathology is that the ways in which radicalized people were identified and categorized, the definitions of radicalization and terrorism, and the methodologies used to assess psychiatric disorders in the diverse populations varied widely across studies.

In addition to the fairly limited empirical literature on women-specific presence of psychopathology, there is also a lack of female-specific data on their role in acts of terrorism or violent extremism based on the findings of our study. The “mixed” data within the present review suggest indications of a contributing role of psychopathology in terrorist or violent extremist behaviors. More specifically, mental health problems (47, 49), cluster B personality traits, i.e., narcissistic and antisocial personality traits (8), and problematic substance use (50) were found to be of influence in the actual proceeding to, or the executing of, such behaviors. Furthermore, based on the “mixed” results, the psychological mechanisms that were found to be at work in terrorist or violent extremist behaviors consisted of experiencing perceived injustice (8), aiming to escape from an intolerable life (47) or feelings of rejection by the community (49).

### 4.1. Limitations and strengths

The current systematic review has some limitations of which the most important one, in our view, is inherent to the major issues in the quality of the current empirical literature on psychopathology in female perpetrators of terrorism and violent extremism. First and for most, because of the small number of subjects in the included studies, the different ways in which psychopathology was assessed and recorded, the varying focus on different aspects of psychopathology, and the diverse terminology used for it, it is difficult to aggregate the results of the included studies. The objectives of our systematic review were not to necessarily provide prevalence rates of psychopathology or data on its potential contributing role in terrorist or violent extremist behaviors, but rather to examine what empirical data on these topics were available. Therefore, it should be noted that due to the nature of the existing literature the review cannot contribute in a meaningful way to a better empirically based understanding of psychopathology among women who exhibit terrorist or violent extremist behaviors. We as authors therefore emphasize interpreting the findings from the present review with caution. Having made this point, and precisely because of the insights gained from the current systematic review, we nevertheless stress the importance of this systematic review as a key observation that endorses a starting position and a call for more future empirical research on the presence and relevance of psychopathology in women with regard to terrorist or violent extremist behaviors. A second limitation may consist in the use of artificial intelligence, and our chosen search strategy within the used artificial intelligence software program. Although this choice was made on a well-founded hypothesis, relevant hits for this systematic review could have been missed since not all records were screened. The risk of missing relevant records, however, also exists when screening is done manually (45). Third, studies were included in case it was clearly described that female participants were part of the study group. Consequently, studies including female participants might have been accidentally missed in case a clear description of the study population was not given. Fourth, publications in languages other than English, Dutch, French, and German have been excluded, which means that relevant studies in other languages might have been missed.

This systematic review also has strengths. First, to the best of our knowledge, this is the first systematic review that takes female perpetrators of terrorist and violent extremist acts as the central study population and investigates the available empirical evidence on psychopathology within this understudied population. Second, the very focus on psychopathology has been broadened by not only including studies reporting on its presence, but also on the contributing role psychopathology potentially plays in terrorist or violent extremist behaviors. Third, by focusing on offenders of terrorism and violent extremism rather than also focusing on radicalized individuals, we have taken into account the potential underlying differences in presence and the potential contributing role of psychopathology between the group that effectively engages in terrorist or violent extremist acts and the group that holds the radical ideas without acting on them. Fourth, only studies based on primary sources have been included in the review making its findings more reliable than when also studies based on secondary data had been included. Fifth, by including mental disorders and pathological personality traits, symptoms or psychological problems, we have prevented an exclusion of studies of individuals in whom psychopathological symptoms were both present and contributed to engaging in the offense, but who have not been diagnosed with a mental disorder due to insufficiently present DSM-criteria to fit a DSM-classification. Sixth, by distinguishing studies based on either terrorist or violent extremist acts, we avoided using these terms interchangeably whilst they differ in their nature as discussed earlier. It should be noted, however, that, given the low numbers of included studies in general, and those with violent extremists as a population in particular, no conclusions could be drawn about potential inter-individual differences in presence and potential contributing role of psychopathology among perpetrators of terrorism vs. violent extremism.

### 4.2. Conclusions and recommendations for future research

Based on our systematic review, we conclude that psychopathology exists in female perpetrators of terrorism and violent extremism and there are indications of it playing a relevant contributing role in their offenses. What was hypothesized a priori, but has now been conclusively proven through this systematic review, is that psychopathology in this population is a largely unexplored field of research. Although our research questions are primarily answered, to be able to contribute in a meaningful way to an understanding of the role of psychopathology as a risk factor in terrorist or violent extremist acts by women, further empirical research is needed. The importance of future research is specially underlined given the more active role of women as perpetrators of terrorism and violent extremism, for example featuring noticeably in the Islamic State. Minimizing the risk of repeated terrorist or violent extremist behaviors by women, along with primary prevention of such acts by radicalized women, requires individualized, i.e., gender-specific, treatments and interventions that address psychopathology as a potential risk factor.

To gain more knowledge about prevalence and contributing role of psychopathology in female perpetrators of terrorism and violent extremism our review also revealed some important starting points for future research. It is important for research on female offenders of terrorism or violent extremism to be based on primary data sources, i.e., psychological or psychiatric assessments, and to examine prevalence rates of mental disorders in addition to pathological personality traits, symptoms and psychopathological problems. In addition, it needs to be studied to what extent and through which psychic mechanisms psychopathology plays a role in terrorist or violent extremist behaviors by women. In this context, our review suggests to, for example, examine whether certain characteristics from a mental disorder, e.g., identity problems arising from cluster B personality problems, are more likely to play a significant role in the actions of this group of female perpetrators. Furthermore, it would be relevant in future research to make a comparison with male perpetrators of terrorism or violent extremism in order to investigate whether gender differences in psychopathology exist, and if so, that attention is paid to these differences in the shaping of counter-terrorism interventions. We expect that through gender-specific management of risk factors, including psychopathology, we can achieve risk minimization of (repeated) terrorist or violent extremist behaviors.

## Data availability statement

The original contributions presented in the study are included in the article/Supplementary material, further inquiries can be directed to the corresponding author.

## Author contributions

SR and DB performed the literature search, screened the references, and synthesized the data. SR wrote the article. MK, ND, and R-JV contributed to the theoretical building of the paper and supervised all the work, read, and approved the manuscript. All authors contributed to the article and approved the submitted version.
