# Supplementary material for: Psychopathology in female offenders of terrorism and violent extremism: a systematic review
Source: Front Psychiatry. 2023 Jun 27;14:1123243. doi: 10.3389/fpsyt.2023.1123243 (PMC10333484; doi:10.3389/fpsyt.2023.1123243)
Supplement: Supplementary file 1 [file Data_Sheet_1.pdf]

Supplementary Material

1 Supplementary Data

1.1 Search Terms

For both literature searches on September 28, 2021, and May 30, 2022, the following search terms were used in title, abstract, and keywords fields:

(Terrorism OR Bioterrorism OR Jihadism OR Violent extremism) AND (Disorders OR Psychopathology OR General Psychiatry OR General Psychology OR Transcultural psychiatry OR Mental disorders OR Intellectual disability OR Narcism OR Identity disorders OR Borderline OR Hysteria OR Psychosis and schizophrenia OR Delinquent and criminal behaviour OR Aggressive and violent behaviour OR Antisocial behaviour OR Addiction and drugs abuse OR Trauma OR PTSD OR Diagnosis (to retrieve disorders) OR Anxiety disorders OR Bipolar disorders OR Cyclothymia OR Depression OR Disruptive behaviour OR Oppositional defiant disorder OR Explosive behaviour OR Fire setting OR Dissociation OR ADHD OR Paraphilia OR Obsessive-compulsive disorder OR Paranoia OR Avoidant personality disorder OR Dependent personality disorder OR Social acceptance OR Emotional regulation OR Fear of rejection OR Impulsivity OR Influenceability OR Rigidity OR Self-esteem OR Self-knowledge OR Sense of emptiness OR Suicidal and self-destructive behaviour) AND (Empirical studies OR Clinical studies OR Case reports)

The entire search strategy and all databases can be requested from the corresponding author.

1.2 Rationale behind Screening Strategy

The a priori hypothesis, i.e., that there were no large numbers of publications that would meet our inclusion criteria, was based on the following two reasons.

Firstly, it was based on the findings of an earlier (unpublished) literature search by the authors of the present systematic review which made it clear that few empirical studies on psychopathology in female terrorism or violent extremism offenders had been published. This search, conducted on October 27, 2020 was performed in the same databases as in the current review and looked for literature on presence and relevance of psychopathology within male and female terrorism and violent extremism offenders. No exclusion criterion regarding gender was thus formulated. ASReview, which also was used at that time, was trained by putting in as prior knowledge a 'golden abstract' that was written by the authors. This abstract represented an ‘ideal’ study, which met the inclusion criteria, i.e., an original study in adult male or female offenders of terrorism or violent extremism in whom prevalence or relevance of psychopathology was directly studied by medical professionals or trained staff. After training ASReview by using this abstract as well as by putting in “relevant” and “irrelevant” references in this software program, the screening phase started. After screening almost 4.000 (out of approximately 20.000) references based on title and abstract, only few studies emerged that included women as participants. Of these studies, none reported women-specific results.

Next to this first reason for forming the aforementioned hypothesis, a second reason was based on the results from the reviews by Gill and colleagues (2021) and Trimbur and colleagues (2021). In these reviews, which looked at the overall population of terrorists and violent extremists, i.e., with no exclusion criterion regarding gender, there were only few studies including women, of which none distinguished its results regarding men and women.

Furthermore, the specific screening strategy for identifying studies via ASReview, i.e., consisting of three rounds with each round having its own active learning model as well as the stopping rule, was constructed in consultation with dr. Rens van de Schoot, professor at Utrecht University, who is an expert on Bayesian statistics and the developer of active learning software for systematic reviewing. Through this strategy, it was made possible not to screen all (more than 20.000) references, but to screen the references with the highest probability of relevance.

1.3 Detailed Description of ASReview Learning Models

The following learning models in ASReview were used for the initial (2021) and the second (2022) search. In the first screening round, we relied on the software's default settings; that is, TF-IDF was selected as the feature extraction technique and Naïve Bayes as the classifier. To avoid local convergence, for the second round, we applied a different feature extraction technique (Doc2Vec) and classifier (Logistic Regression), and we used the labelling decisions from the first round for the second round as training data. Because we expected concept ambiguity, for the third round, we applied sentence BERT and a fully connected neural network, again using all previous labelling decisions as training data. By applying these three different models, we could be more certain to have captured all potentially relevant papers whilst screening references via ASReview.

1.4 Assessment of risk of bias in included studies according to SIGN

For the assessment of risk of bias in included studies The Scottish Intercollegiate Guidelines Network (SIGN) framework was used. This framework encompasses the following levels of evidence: 1++ – ‘High quality meta-analyses, systematic reviews of RCTs, or RCTs with a very low risk of bias’, 1+ – ‘Well-conducted meta-analyses, systematic reviews, or RCTs with a low risk of bias’, 1− – ‘Meta-analyses, systematic reviews, or RCTs with a high risk of bias’, 2++ – ‘High quality systematic reviews of case control or cohort or studies’, 2+ – Well conducted case control or cohort studies with a low risk of confounding or bias and a moderate probability that the relationship is causal”, 2− – ‘Case control or cohort studies with a high risk of confounding or bias and a significant risk that the relationship is not causal’, 3 – ‘Non-analytic studies, such as case reports or case series’, and 4 – ‘Expert Opinion’ (‘SIGN 50’, 2011).
